# Supplementary figures and images for: Pediatric Long COVID Subphenotypes: An EHR-based study from the RECOVER program
Source: PLOS Digit Health. 2025 Apr 10;4(4):e0000747. doi: 10.1371/journal.pdig.0000747 (PMC11984710; doi:10.1371/journal.pdig.0000747)

**S1 Fig: Heatmap of incident post-acute diagnoses by subphenotype, Cohort A**

**
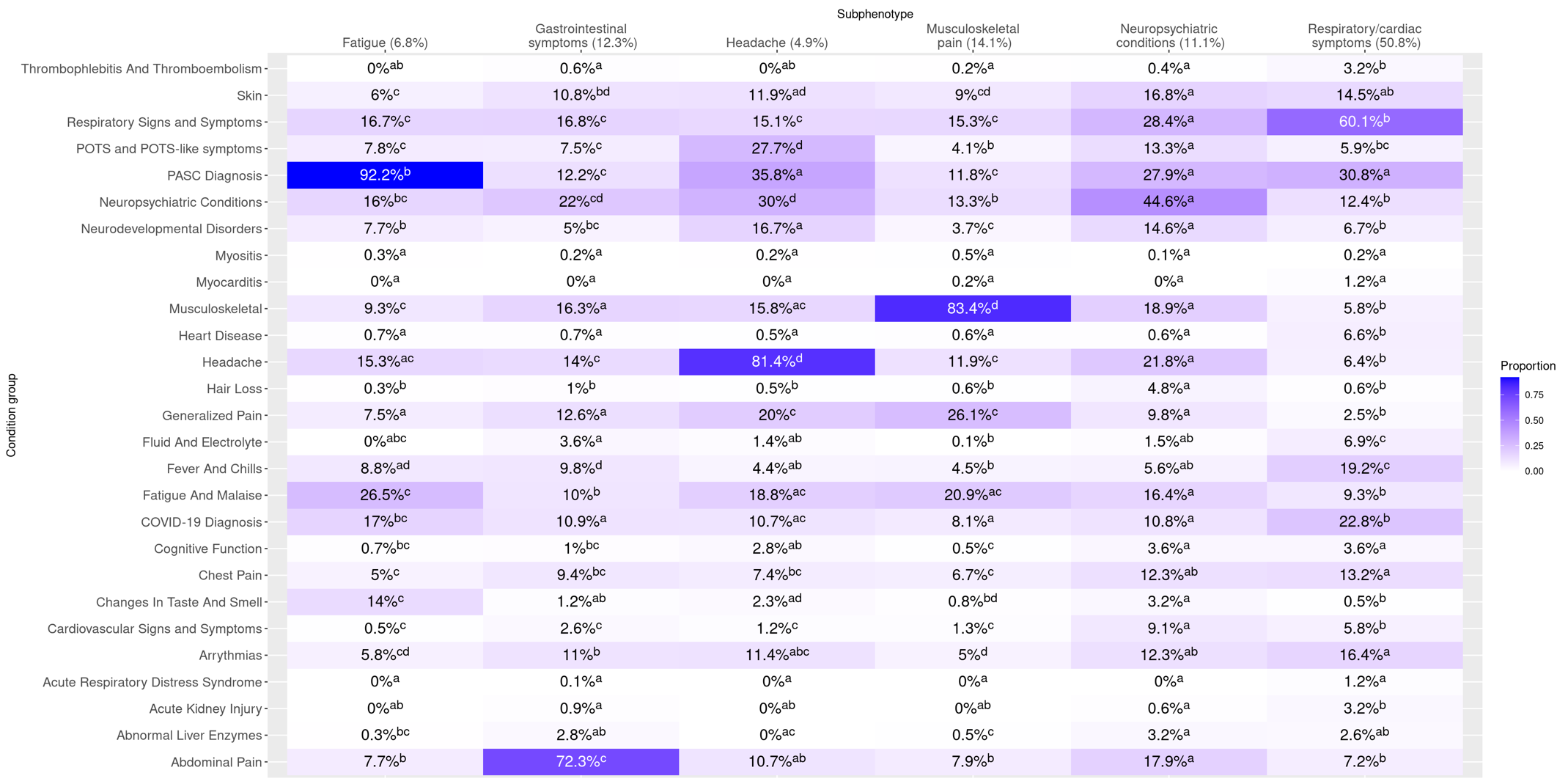
**

Supplement: S1 Fig — (DOCX) [file pdig.0000747.s001.docx]

**S2 Fig: Heatmap of incident post-acute diagnoses by cluster, Cohort A**

**
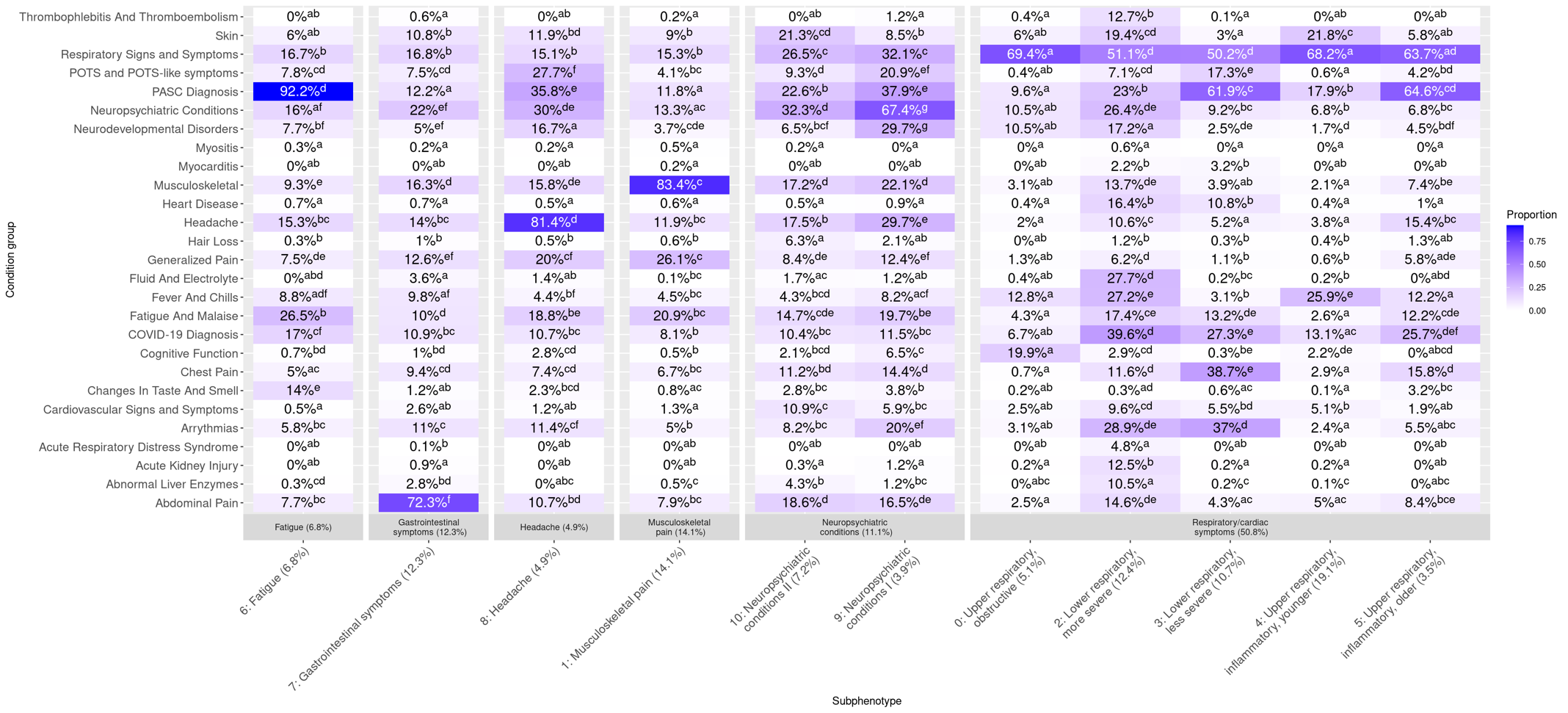
**

Supplement: S2 Fig — (DOCX) [file pdig.0000747.s002.docx]

**S3 Fig: Heatmap of incident post-acute diagnoses by cluster, Cohort B**

**
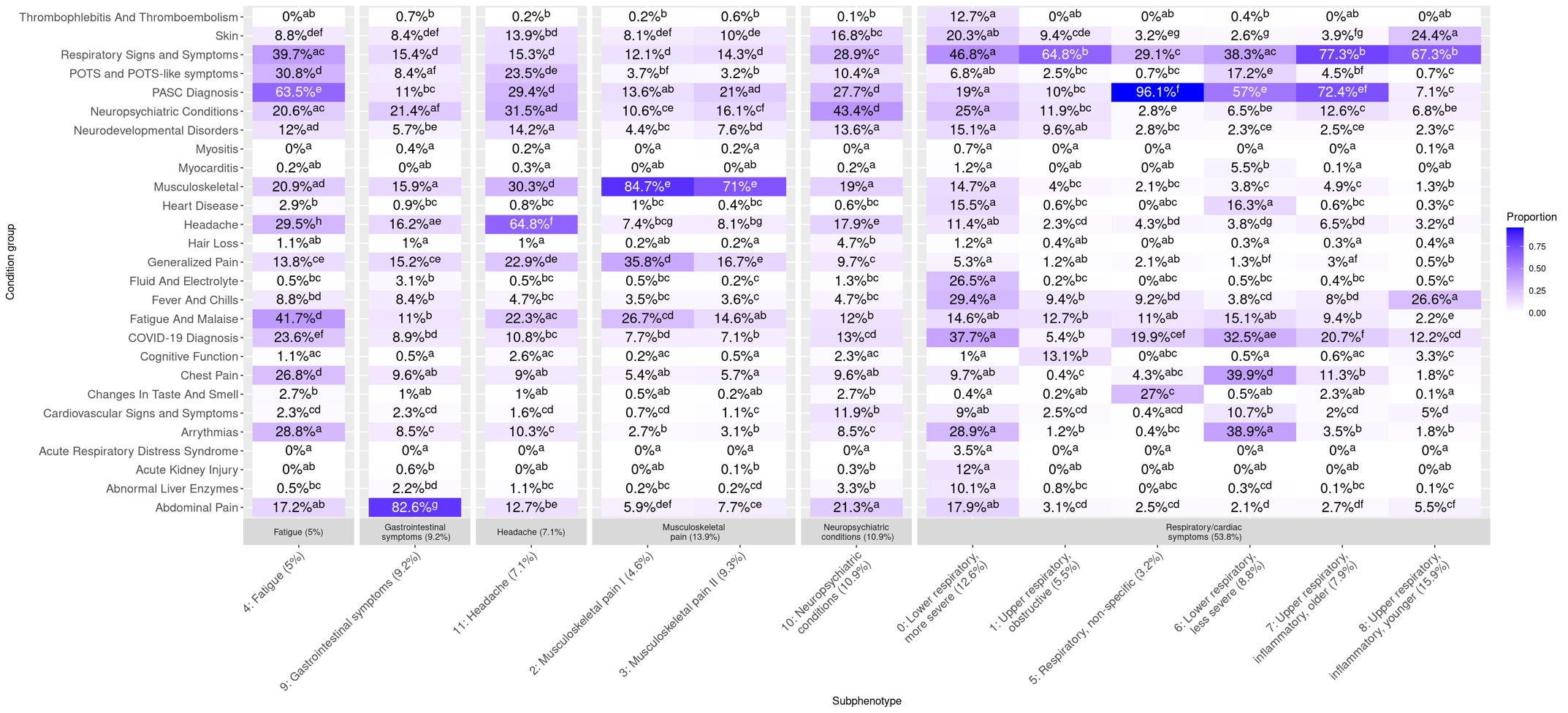
**

Supplement: S3 Fig — (DOCX) [file pdig.0000747.s003.docx]

**S4 Fig: Healthcare utilization trajectories, cohort B**

**
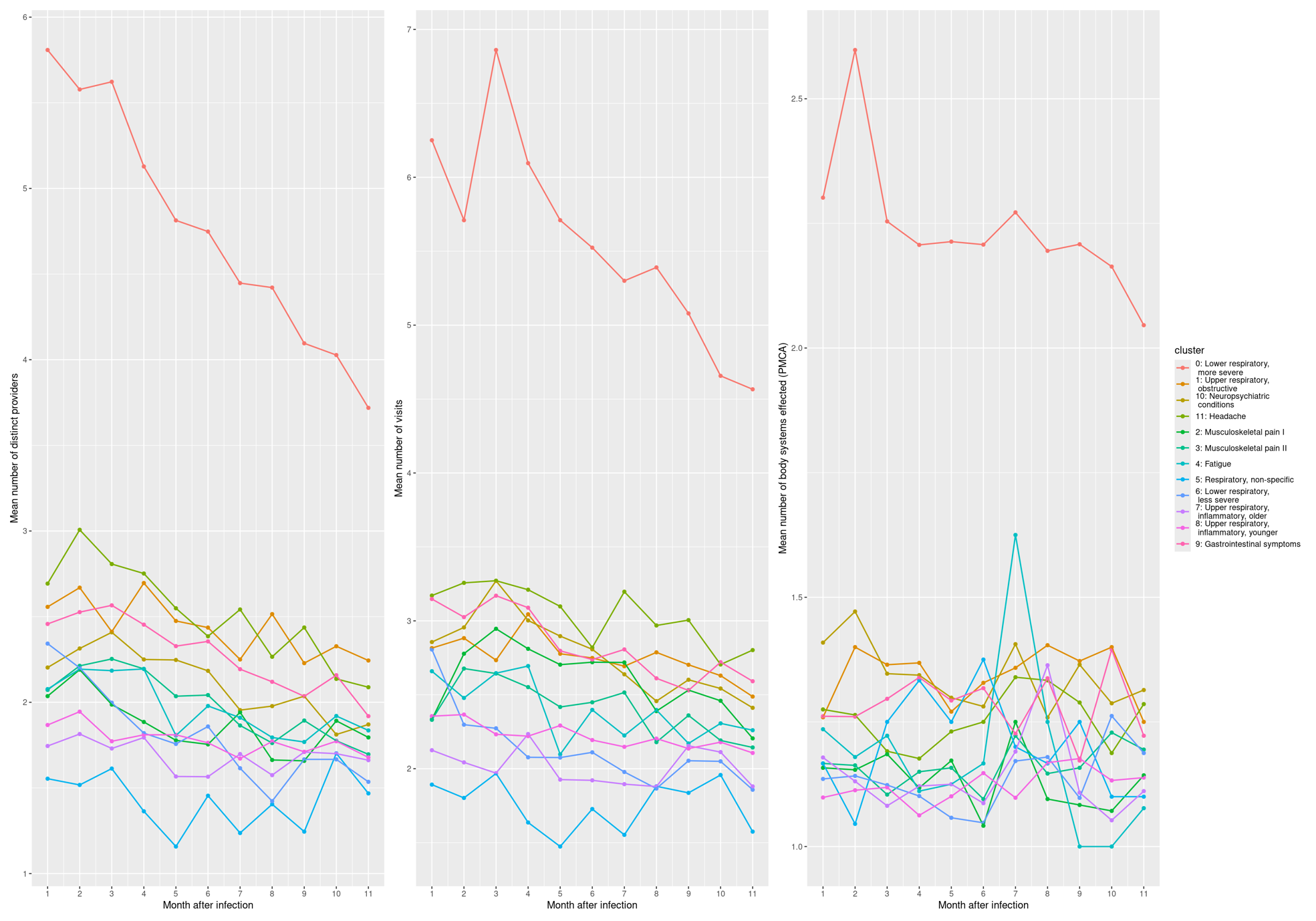
**

Supplement: S4 Fig — (DOCX) [file pdig.0000747.s004.docx]

**S5 Fig: Heatmap of pre-existing chronic conditions by cluster, cohort B**

**
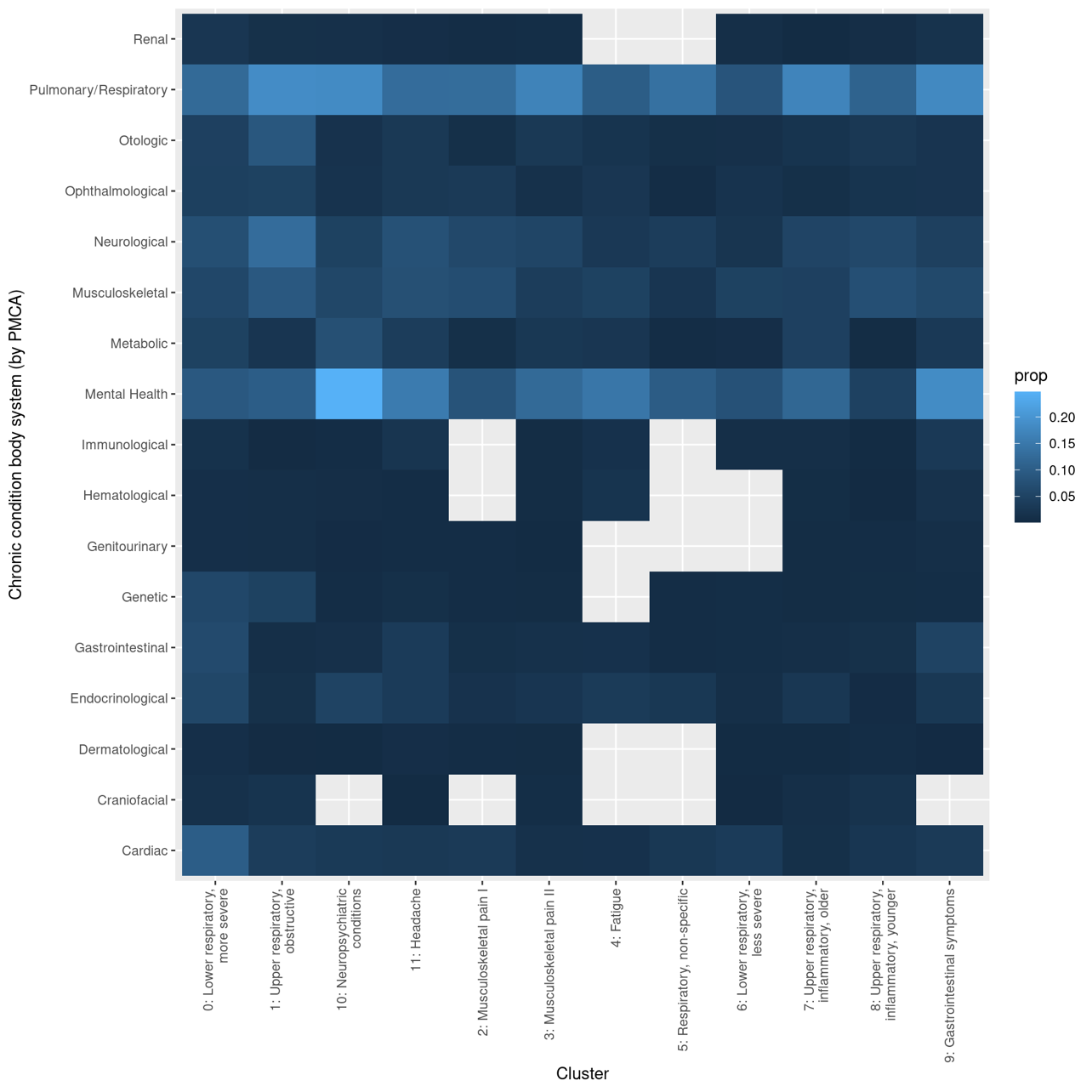
**

Supplement: S5 Fig — (DOCX) [file pdig.0000747.s005.docx]

**S6 Fig: Heatmap of incident follow-up diagnoses for matched control cohort**

**
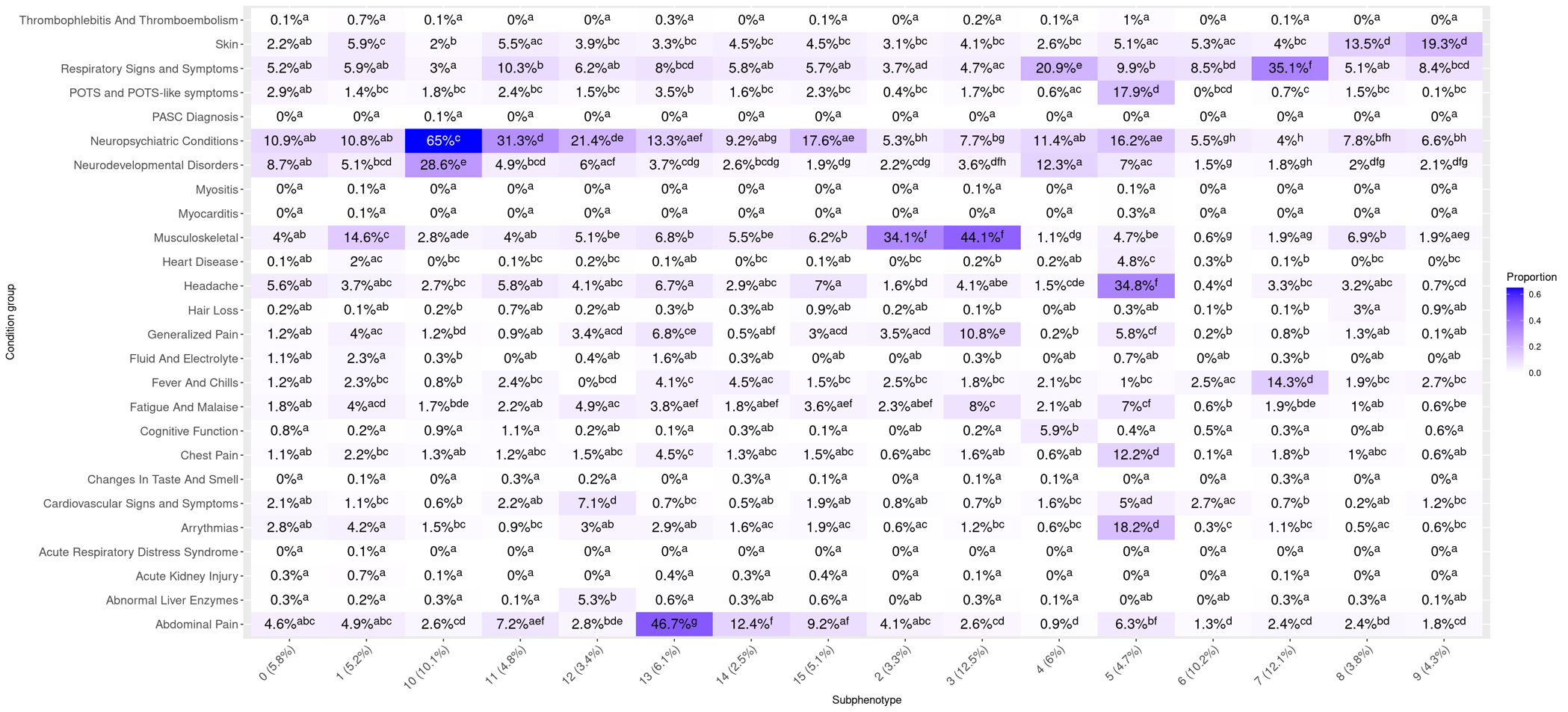
**

Supplement: S6 Fig — (DOCX) [file pdig.0000747.s006.docx]

**S7 Fig: Subphenotype centroids: comparison of cohort A, cohort B, and matched control cohort.**

**
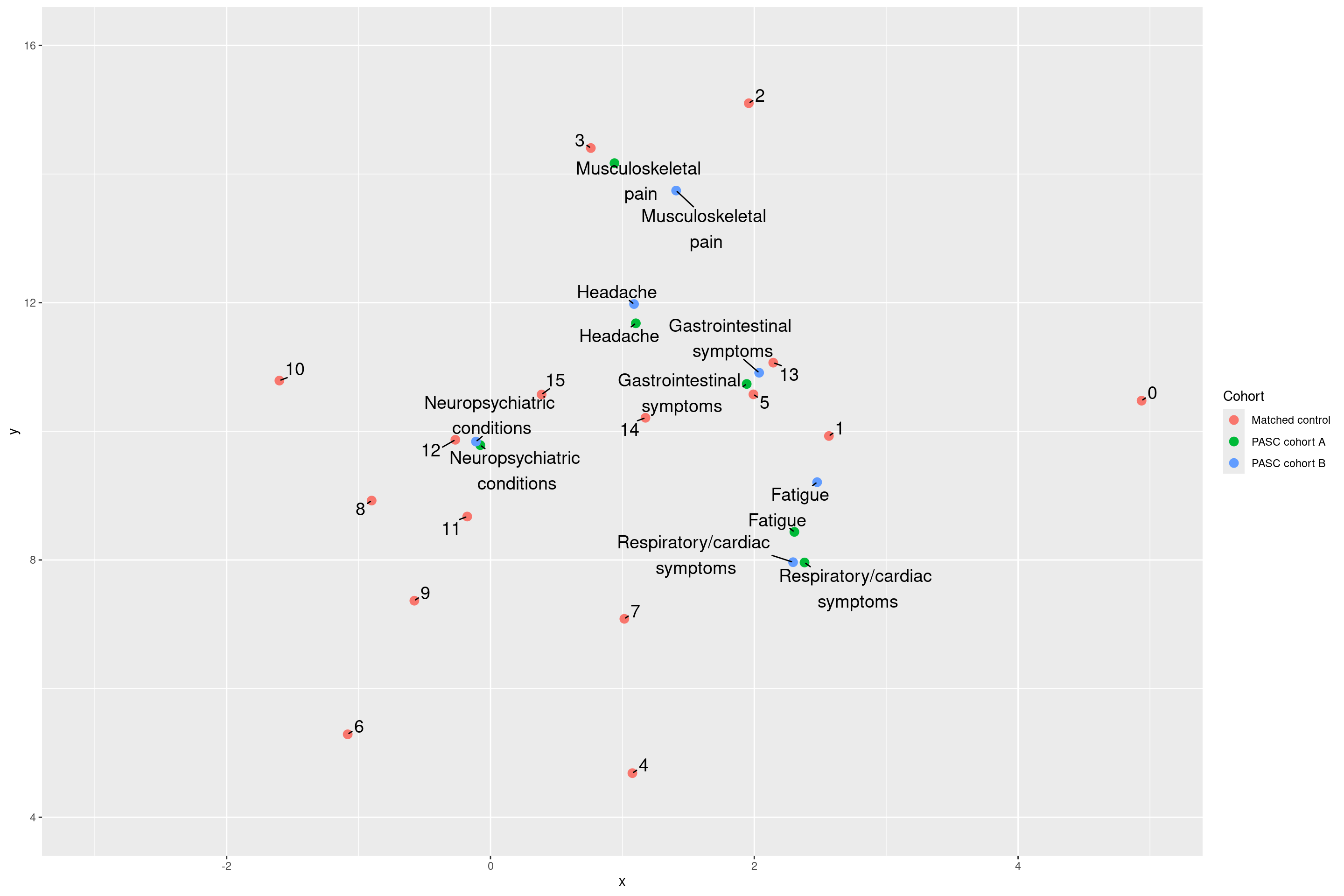
**

Supplement: S7 Fig — (DOCX) [file pdig.0000747.s007.docx]
