# Supplementary material for: Pediatric Long COVID Subphenotypes: An EHR-based study from the RECOVER program
Source: PLOS Digit Health. 2025 Apr 10;4(4):e0000747. doi: 10.1371/journal.pdig.0000747 (PMC11984710; doi:10.1371/journal.pdig.0000747)
Supplement: S8 Fig — (DOCX) [file pdig.0000747.s008.docx]

**S8 Fig: Heatmap of incident post-acute diagnoses by cluster, with unclustered patients shown separately, cohort B**

**
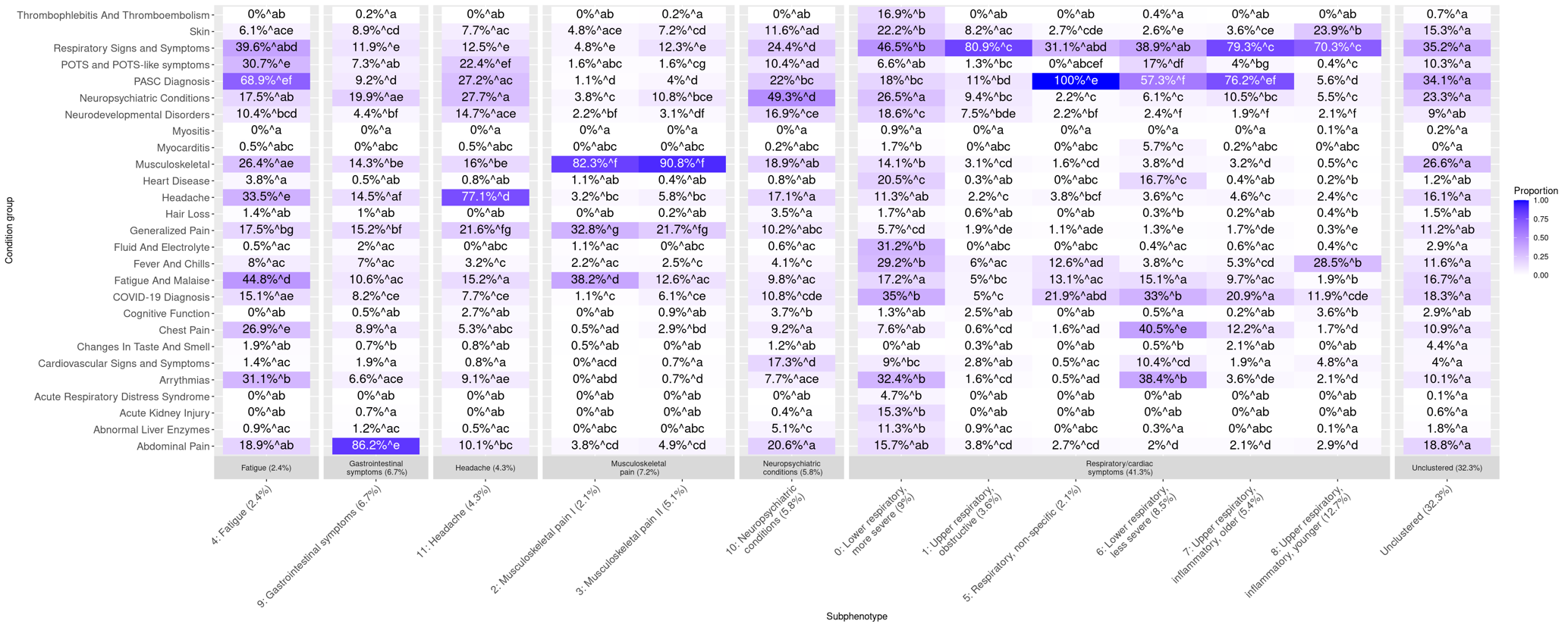
**
