# Supplementary material for: Pediatric Long COVID Subphenotypes: An EHR-based study from the RECOVER program
Source: PLOS Digit Health. 2025 Apr 10;4(4):e0000747. doi: 10.1371/journal.pdig.0000747 (PMC11984710; doi:10.1371/journal.pdig.0000747)
Supplement: S1 Table — (DOCX) [file pdig.0000747.s009.docx]

**S1 Table: Demographic and clinical characteristics of clusters, cohort A**

Note: cells marked with an asterisk have been modified by a random count between 0 and 4 to prevent reidentification of that cell or a cell in the same group.

| **Subphenotype** |  | Fatigue (600, 6.8%) | Gastrointestinal symptoms (1078, 12.3%) | Headache (430, 4.9%) | Musculoskeletal pain (1232, 14.1%) | Neuropsychiatric conditions (974, 11.1%) | | Respiratory/cardiac symptoms (4454, 50.8%) | | | | |
| --- | --- | --- | --- | --- | --- | --- | --- | --- | --- | --- | --- | --- |
| **Cluster** |  | 6: Fatigue (600, 6.8%) | 7: Gastrointestinal symptoms (1078, 12.3%) | 8: Headache (430, 4.9%) | 1: Musculoskeletal pain (1232, 14.1%) | 9: Neuropsychiatric conditions I (340, 3.9%) | 10: Neuropsychiatric conditions II (634, 7.2%) | 0: Upper respiratory, obstructive (447, 5.1%) | 2: Lower respiratory, more severe (1089, 12.4%) | 3: Lower respiratory, less severe (935, 10.7%) | 4: Upper respiratory, inflammatory, younger (1672, 19.1%) | 5: Upper respiratory, inflammatory, older (311, 3.5%) |
| **Age group (n/%)** | <1 | 5 (0.8%) | 13 (1.2%) | 0 (0%) | 16 (1.3%) | 0 (0%) | 0 (0%) | 72 (16.1%) | 258 (23.7%) | 18 (1.9%) | 488 (29.2%) | 2 (0.1%)* |
|  | 1-4 | 45 (7.5%) | 73 (6.8%) | 3 (0.7%)* | 58 (4.7%) | 5 (1.5%)* | 30 (4.7%) | 241 (53.9%) | 216 (19.8%) | 35 (3.7%) | 626 (37.4%) | 15 (4.8%)* |
|  | 5-11 | 157 (26.2%) | 280 (26%) | 96 (22.3%)* | 284 (23.1%) | 66 (19.4%)* | 142 (22.4%) | 104 (23.3%) | 154 (14.1%) | 164 (17.5%) | 378 (22.6%) | 75 (24.1%) |
|  | 12-15 | 185 (30.8%) | 275 (25.5%) | 154 (35.8%) | 410 (33.3%) | 104 (30.6%) | 194 (30.6%) | 13 (2.9%) | 160 (14.7%) | 302 (32.3%) | 93 (5.6%) | 73 (23.5%) |
|  | 16-20 | 208 (34.7%) | 437 (40.5%) | 177 (41.2%) | 464 (37.7%) | 165 (48.5%) | 268 (42.3%) | 17 (3.8%) | 301 (27.6%) | 416 (44.5%) | 87 (5.2%) | 146 (46.9%) |
| **Sex (n/%)** | Female | 306 (51%) | 722 (67%) | 296 (68.8%) | 661 (53.7%) | 203 (59.7%) | 422 (66.6%) | 179 (40%) | 546 (50.1%) | 511 (54.7%) | 762 (45.6%) | 169 (54.3%) |
|  | Male/Other/Unknown | 294 (49%) | 356 (33%) | 134 (31.2%) | 571 (46.3%) | 137 (40.3%) | 212 (33.4%) | 268 (60%) | 543 (49.9%) | 424 (45.3%) | 910 (54.4%) | 142 (45.7%) |
| **Race/ethnicity (n/%)** | Non-Hispanic Black/AA | 41 (6.8%) | 115 (10.7%) | 41 (9.5%) | 176 (14.3%) | 41 (12.1%) | 92 (14.5%) | 71 (15.9%) | 209 (19.2%) | 97 (10.4%) | 255 (15.3%) | 26 (8.4%) |
|  | Non-Hispanic Asian/PI | 14 (2.3%) | 27 (2.5%) | 12 (2.8%) | 36 (2.9%) | 7 (2.1%) | 28 (4.4%) | 12 (2.7%) | 49 (4.5%) | 22 (2.4%) | 93 (5.6%) | 14 (4.5%) |
|  | Hispanic | 97 (16.2%) | 291 (27%) | 67 (15.6%) | 216 (17.5%) | 52 (15.3%) | 211 (33.3%) | 97 (21.7%) | 292 (26.8%) | 157 (16.8%) | 452 (27%) | 73 (23.5%) |
|  | Non-Hispanic White | 372 (62%) | 579 (53.7%) | 270 (62.8%) | 688 (55.8%) | 204 (60%) | 246 (38.8%) | 220 (49.2%) | 439 (40.3%) | 543 (58.1%) | 637 (38.1%) | 174 (55.9%) |
|  | Multiple | 3 (0.5%)* | 12 (1.1%) | 8 (1.9%) | 38 (3.1%) | 9 (2.6%) | 1 (0.2%)* | 13 (2.9%) | 25 (2.3%) | 27 (2.9%) | 47 (2.8%) | 0 (0%) |
|  | Other/Unknown | 73 (12.2%)* | 54 (5%) | 32 (7.4%) | 78 (6.3%) | 27 (7.9%) | 56 (8.8%)* | 34 (7.6%) | 75 (6.9%) | 89 (9.5%) | 188 (11.2%) | 24 (7.7%) |
| **Cohort entry period (n/%)** | Mar-Jun 2020 | 15 (2.5%) | 26 (2.4%) | 6 (1.4%) | 19 (1.5%) | 8 (2.4%) | 15 (2.4%) | 6 (1.3%) | 46 (4.2%) | 13 (1.4%) | 10 (0.6%) | 4 (1.3%)* |
|  | Jul-Oct 2020 | 22 (3.7%) | 70 (6.5%) | 24 (5.6%) | 106 (8.6%) | 24 (7.1%) | 42 (6.6%) | 13 (2.9%) | 81 (7.4%) | 56 (6%) | 45 (2.7%) | 8 (2.6%)* |
|  | Nov-Feb 2021 | 53 (8.8%) | 175 (16.2%) | 53 (12.3%) | 228 (18.5%) | 46 (13.5%) | 103 (16.2%) | 49 (11%) | 119 (10.9%) | 141 (15.1%) | 108 (6.5%) | 17 (5.5%) |
|  | Mar-Jun 2021 | 86 (14.3%) | 60 (5.6%) | 26 (6%) | 83 (6.7%) | 16 (4.7%) | 37 (5.8%) | 20 (4.5%) | 125 (11.5%) | 89 (9.5%) | 73 (4.4%) | 7 (2.3%) |
|  | Jul-Oct 2021 | 104 (17.3%) | 182 (16.9%) | 71 (16.5%) | 215 (17.5%) | 72 (21.2%) | 96 (15.1%) | 55 (12.3%) | 184 (16.9%) | 182 (19.5%) | 236 (14.1%) | 80 (25.7%) |
|  | Nov-Feb 2022 | 188 (31.3%) | 389 (36.1%) | 151 (35.1%) | 427 (34.7%) | 116 (34.1%) | 234 (36.9%) | 205 (45.9%) | 303 (27.8%) | 284 (30.4%) | 692 (41.4%) | 122 (39.2%) |
|  | Mar-Jun 2022 | 95 (15.8%) | 107 (9.9%) | 58 (13.5%) | 86 (7%) | 37 (10.9%) | 72 (11.4%) | 58 (13%) | 134 (12.3%) | 110 (11.8%) | 285 (17%) | 34 (10.9%) |
|  | Jul-Aug 2022 | 37 (6.2%) | 69 (6.4%) | 41 (9.5%) | 68 (5.5%) | 21 (6.2%) | 35 (5.5%) | 41 (9.2%) | 97 (8.9%) | 60 (6.4%) | 223 (13.3%) | 39 (12.5%) |
| **ICU (acute) (n/%)** |  | 0 (0%) | 10 (0.9%) | 0 (0%) | 7 (0.6%) | 2 (0.6%)* | 9 (1.4%) | 3 (0.7%)* | 95 (8.7%) | 14 (1.5%) | 7 (0.4%) | 0 (0%) |
| **Hospitalization (acute) (n/%)** |  | 1 (0.2%)* | 82 (7.6%) | 10 (2.3%) | 52 (4.2%) | 22 (6.5%) | 14 (2.2%) | 26 (5.8%) | 302 (27.7%) | 30 (3.2%) | 57 (3.4%) | 3 (1.0%)* |
| **COVID acute phase severity of illness (n/%)** | Asymptomatic | 525 (87.5%) | 572 (53.1%) | 270 (62.8%) | 752 (61%) | 235 (69.1%) | 403 (63.6%) | 259 (57.9%) | 662 (60.8%) | 733 (78.4%) | 881 (52.7%) | 232 (74.6%) |
|  | Mild | 66 (11%) | 405 (37.6%) | 136 (31.6%) | 417 (33.8%) | 91 (26.8%) | 208 (32.8%) | 143 (32%) | 184 (16.9%) | 149 (15.9%) | 671 (40.1%) | 70 (22.5%) |
|  | Moderate | 8 (1.3%)* | 82 (7.6%) | 16 (3.7%) | 51 (4.1%) | 9 (2.6%) | 15 (2.4%) | 30 (6.7%) | 101 (9.3%) | 19 (2%) | 105 (6.3%) | 9 (2.9%) |
|  | Severe | 1 (0.2%)* | 19 (1.8%) | 8 (1.9%) | 12 (1%) | 5 (1.5%) | 8 (1.3%) | 15 (3.4%) | 142 (13%) | 34 (3.6%) | 15 (0.9%) | 0 (0%) |
| **Presence of existing chronic condition (n/%)** |  | 172 (28.7%) | 496 (46%) | 156 (36.3%) | 445 (36.1%) | 178 (52.4%) | 283 (44.6%) | 189 (42.3%) | 368 (33.8%) | 270 (28.9%) | 556 (33.3%) | 113 (36.3%) |
| **Most common diagnoses** |  | U09.9: Post COVID-19 condition, unspecified (69.7%)  B94.8: Sequelae of other specified infectious and parasitic diseases (27.7%) | R10.9: Unspecified abdominal pain (39.0%)  R10.84: Generalized abdominal pain (26.4%) | R51.9: Headache, unspecified (47.0%)  U09.9: Post COVID-19 condition, unspecified (32.3%) | G89.29: Other chronic pain (22.6%) | U09.9: Post COVID-19 condition, unspecified (30.9%)  F41.9: Anxiety disorder, unspecified (23.2%) | U09.9: Post COVID-19 condition, unspecified (20.5%) | R06.83: Snoring (40.9%)  R09.81: Nasal congestion (25.3%)  G47.30: Sleep apnea, unspecified (21.5%) | U07.1: Emergency use of U07.1 \| COVID-19 (38.8%)  R50.9: Fever, unspecified (22.2%) | U09.9: Post COVID-19 condition, unspecified (52.9%)  R07.9: Chest pain, unspecified (29.6%)  R06.02: Shortness of breath (29.4%) U07.1: Emergency use of U07.1 \| COVID-19 (27.3%)  R00.2: Palpitations (20.1%) | R05.9: Cough, unspecified (34.03%)  R50.9: Fever, unspecified (24.2%)  R09.81: Nasal congestion (21.2%) | U09.9: Post COVID-19 condition, unspecified (62.1%)  R05.9: Cough, unspecified (30.9%)  U07.1: Emergency use of U07.1 \| COVID-19 (25.4%) |
