# Supplementary material for: Pediatric Long COVID Subphenotypes: An EHR-based study from the RECOVER program
Source: PLOS Digit Health. 2025 Apr 10;4(4):e0000747. doi: 10.1371/journal.pdig.0000747 (PMC11984710; doi:10.1371/journal.pdig.0000747)
Supplement: S2 Table — (DOCX) [file pdig.0000747.s010.docx]

**S2 Table: Demographic and clinical characteristics of clusters, cohort B**

Note: cells marked with an asterisk have been modified by a random count between 0 and 4 to prevent reidentification of that cell or a cell in the same group.

| **Subphenotype** |  | Fatigue (441, 5%) | Gastrointestinal symptoms (810, 9.2%) | Headache (620, 7.1%) | Musculoskeletal pain (1218, 13.9%) | | Neuropsychiatric conditions (952, 10.9%) | Respiratory/cardiac symptoms (4716, 53.8%) | | | | | |
| --- | --- | --- | --- | --- | --- | --- | --- | --- | --- | --- | --- | --- | --- |
| **Cluster** |  | 4: Fatigue (441, 5%) | 9: Gastrointestinal symptoms (810, 9.2%) | 11: Headache (620, 7.1%) | 2: Musculoskeletal pain I (405, 4.6%) | 3: Musculoskeletal pain II (813, 9.3%) | 10: Neuropsychiatric conditions (952, 10.9%) | 0: Lower respiratory, more severe (1106, 12.6%) | 1: Upper respiratory, obstructive (480, 5.5%) | 5: Respiratory, non-specific (282, 3.2%) | 6: Lower respiratory, less severe (767, 8.8%) | 7: Upper respiratory, inflammatory, older (691, 7.9%) | 8: Upper respiratory, inflammatory, younger (1390, 15.9%) |
| **Age group (n/%)** | <1 | 0 (0%) | 6 (0.7%) | 0 (0%) | 10 (2.5%) | 7 (0.9%) | 4 (0.4%)* | 254 (23%) | 94 (19.6%) | 9 (3.2%) | 20 (2.6%) | 11 (1.6%) | 428 (30.8%) |
|  | 1-4 | 3 (0.7%)* | 49 (6%) | 16 (2.6%) | 11 (2.7%) | 49 (6%) | 21 (2.2%)* | 197 (17.8%) | 239 (49.8%) | 29 (10.3%) | 31 (4%) | 71 (10.3%) | 595 (42.8%) |
|  | 5-11 | 89 (20.2%)* | 233 (28.8%) | 116 (18.7%) | 78 (19.3%) | 237 (29.2%) | 190 (20%) | 182 (16.5%) | 95 (19.8%) | 69 (24.5%) | 144 (18.8%) | 185 (26.8%) | 303 (21.8%) |
|  | 12-15 | 145 (32.9%) | 206 (25.4%) | 233 (37.6%) | 156 (38.5%) | 263 (32.3%) | 298 (31.3%) | 160 (14.5%) | 35 (7.3%) | 88 (31.2%) | 249 (32.5%) | 175 (25.3%) | 39 (2.8%) |
|  | 16-20 | 204 (46.3%) | 316 (39%) | 255 (41.1%) | 150 (37%) | 257 (31.6%) | 439 (46.1%) | 313 (28.3%) | 17 (3.5%) | 87 (30.9%) | 323 (42.1%) | 249 (36%) | 25 (1.8%) |
| **Sex (n/%)** | Female | 285 (64.6%) | 536 (66.2%) | 421 (67.9%) | 225 (55.6%) | 385 (47.4%) | 643 (67.5%) | 576 (52.1%) | 201 (41.9%) | 149 (52.8%) | 383 (49.9%) | 367 (53.1%) | 607 (43.7%) |
|  | Male/Other/Unknown | 156 (35.4%) | 274 (33.8%) | 199 (32.1%) | 180 (44.4%) | 428 (52.6%) | 309 (32.5%) | 530 (47.9%) | 279 (58.1%) | 133 (47.2%) | 384 (50.1%) | 324 (46.9%) | 783 (56.3%) |
| **Race/ethnicity (n/%)** | Non-Hispanic Black/AA | 43 (9.8%) | 105 (13%) | 73 (11.8%) | 52 (12.8%) | 125 (15.4%) | 139 (14.6%) | 197 (17.8%) | 90 (18.8%) | 30 (10.6%) | 76 (9.9%) | 67 (9.7%) | 233 (16.8%) |
|  | Non-Hispanic Asian/PI | 11 (2.5%) | 19 (2.3%) | 6 (1%) | 9 (2.2%) | 13 (1.6%) | 29 (3%) | 45 (4.1%) | 16 (3.3%) | 7 (2.5%) | 18 (2.3%) | 19 (2.7%) | 64 (4.6%) |
|  | Hispanic | 69 (15.6%) | 183 (22.6%) | 100 (16.1%) | 66 (16.3%) | 127 (15.6%) | 251 (26.4%) | 305 (27.6%) | 102 (21.2%) | 59 (20.9%) | 129 (16.8%) | 150 (21.7%) | 410 (29.5%) |
|  | Non-Hispanic White | 264 (59.9%) | 426 (52.6%) | 368 (59.4%) | 243 (60%) | 467 (57.4%) | 457 (48%) | 447 (40.4%) | 236 (49.2%) | 145 (51.4%) | 446 (58.1%) | 363 (52.5%) | 502 (36.1%) |
|  | Multiple | 9 (2%) | 18 (2.2%) | 16 (2.6%) | 11 (2.7%) | 23 (2.8%) | 15 (1.6%) | 27 (2.4%) | 16 (3.3%) | 6 (2.1%) | 25 (3.3%) | 12 (1.7%) | 27 (1.9%) |
|  | Other/Unknown | 45 (10.2%) | 59 (7.3%) | 57 (9.2%) | 24 (5.9%) | 58 (7.1%) | 61 (6.4%) | 85 (7.7%) | 20 (4.2%) | 35 (12.4%) | 73 (9.5%) | 80 (11.6%) | 154 (11.1%) |
| **Cohort entry period (n/%)** | Mar-Jun 2020 | 2 (0.7%)* | 21 (2.6%) | 10 (1.6%) | 7 (1.7%) | 37 (4.6%) | 19 (2%) | 50 (4.5%) | 12 (2.5%) | 2 (0.7%)* | 19 (2.5%) | 3 (0.4%) | 7 (0.5%) |
|  | Jul-Oct 2020 | 19 (4.3%)* | 50 (6.2%) | 38 (6.1%) | 22 (5.4%) | 86 (10.6%) | 52 (5.5%) | 71 (6.4%) | 18 (3.8%) | 1 (0.4%)* | 38 (5%) | 14 (2%)* | 27 (1.9%) |
|  | Nov-Feb 2021 | 47 (10.7%) | 148 (18.3%) | 102 (16.5%) | 79 (19.5%) | 160 (19.7%) | 153 (16.1%) | 170 (15.4%) | 55 (11.5%) | 12 (4.3%) | 120 (15.6%) | 40 (5.8%) | 111 (8%) |
|  | Mar-Jun 2021 | 33 (7.5%) | 50 (6.2%) | 43 (6.9%) | 27 (6.7%) | 87 (10.7%) | 62 (6.5%) | 120 (10.8%) | 27 (5.6%) | 13 (4.6%) | 91 (11.9%) | 29 (4.2%) | 57 (4.1%) |
|  | Jul-Oct 2021 | 79 (17.9%) | 113 (14%) | 102 (16.5%) | 67 (16.5%) | 105 (12.9%) | 147 (15.4%) | 162 (14.6%) | 53 (11%) | 77 (27.3%) | 138 (18%) | 132 (19.1%) | 199 (14.3%) |
|  | Nov-Feb 2022 | 155 (35.1%) | 295 (36.4%) | 220 (35.5%) | 143 (35.3%) | 247 (30.4%) | 346 (36.3%) | 304 (27.5%) | 213 (44.4%) | 104 (36.9%) | 235 (30.6%) | 255 (36.9%) | 567 (40.8%) |
|  | Mar-Jun 2022 | 59 (13.4%) | 79 (9.8%) | 59 (9.5%) | 30 (7.4%) | 59 (7.3%) | 112 (11.8%) | 124 (11.2%) | 58 (12.1%) | 55 (19.5%) | 86 (11.2%) | 138 (20%) | 230 (16.5%) |
|  | Jul-Aug 2022 | 47 (10.7%) | 54 (6.7%) | 46 (7.4%) | 30 (7.4%) | 32 (3.9%) | 61 (6.4%) | 105 (9.5%) | 44 (9.2%) | 18 (6.4%) | 40 (5.2%) | 80 (11.6%) | 192 (13.8%) |
| **ICU (acute) (n/%)** |  | 0 (0%) | 2 (0.2%)* | 5 (0.8%) | 4 (1.0%)* | 6 (0.7%) | 6 (0.6%) | 118 (10.7%) | 3 (0.6%)* | 0 (0%) | 13 (1.7%) | 3 (0.4%)* | 1 (0.1%)* |
| **Hospitalization (acute) (n/%)** |  | 4 (0.9%)* | 56 (6.9%) | 21 (3.4%) | 7 (1.7%) | 33 (4.1%) | 34 (3.6%) | 303 (27.4%) | 33 (6.9%) | 0 (0%) | 40 (5.2%) | 7 (1%) | 42 (3%) |
| **COVID acute phase severity of illness (n/%)** | Asymptomatic | 336 (76.2%) | 451 (55.7%) | 404 (65.2%) | 261 (64.4%) | 499 (61.4%) | 624 (65.5%) | 654 (59.1%) | 261 (54.4%) | 256 (90.8%) | 584 (76.1%) | 564 (81.6%) | 675 (48.6%) |
|  | Mild | 89 (20.2%) | 287 (35.4%) | 184 (29.7%) | 123 (30.4%) | 269 (33.1%) | 290 (30.5%) | 177 (16%) | 173 (36%) | 23 (8.2%)* | 130 (16.9%) | 113 (16.4%) | 607 (43.7%) |
|  | Moderate | 11 (2.5%) | 62 (7.7%) | 23 (3.7%) | 16 (4%) | 33 (4.1%) | 28 (2.9%) | 89 (8%) | 39 (8.1%) | 3 (1.1%)* | 18 (2.3%) | 9 (1.3%) | 89 (6.4%) |
|  | Severe | 5 (1.1%) | 10 (1.2%) | 9 (1.5%) | 5 (1.2%) | 12 (1.5%) | 10 (1.1%) | 186 (16.8%) | 7 (1.5%) | 0 (0%) | 35 (4.6%) | 5 (0.7%) | 19 (1.4%) |
| **Presence of existing chronic condition (n/%)** |  | 160 (36.3%) | 373 (46%) | 250 (40.3%) | 161 (39.8%) | 297 (36.5%) | 428 (45%) | 384 (34.7%) | 221 (46%) | 81 (28.7%) | 198 (25.8%) | 250 (36.2%) | 462 (33.2%) |
| **Most common diagnoses** |  | U09.9: Post COVID-19 condition, unspecified (57.6%)  R53.83: Other fatigue (30.8%)  U07.1: Emergency use of U07.1 \| COVID-19 (23.4%) R51.9: Headache, unspecified (23.1%)  R42: Dizziness and giddiness (22.2%) | R10.9: Unspecified abdominal pain (45.6%)  R10.84: Generalized abdominal pain (28.8%)  R10.13: Epigastric pain (21.2%)  K59.00: Constipation, unspecified (21.2%) | R51.9: Headache, unspecified (37.9%)  U09.9: Post COVID-19 condition, unspecified (25.5%) | G89.29: Other chronic pain (32.4%)  M62.81: Muscle weakness (generalized) (20.5%)  M54.50: Low back pain, unspecified (20.0%) |  | U09.9: Post COVID-19 condition, unspecified (23.9%) | U07.1: Emergency use of U07.1 \| COVID-19 (36.9%)  R50.9: Fever, unspecified (25.4%) | R06.83: Snoring (36.3%)  R09.81: Nasal congestion (21.5%) | U09.9: Post COVID-19 condition, unspecified (92.6%) | U09.9: Post COVID-19 condition, unspecified (46.2%)  U07.1: Emergency use of U07.1 \| COVID-19 (32.5%)  R07.9: Chest pain, unspecified (31.0%)  R06.02: Shortness of breath (23.2%)  B94.8: Sequelae of other specified infectious and parasitic diseases (20.6%) | U09.9: Post COVID-19 condition, unspecified (70.0%)  R05.3: Chronic cough (30.97%)  R05.9: Cough, unspecified (26.0%)  U07.1: Emergency use of U07.1 \| COVID-19 (20.7%) | R05.9: Cough, unspecified (33.2%)  R50.9: Fever, unspecified (25.2%)  R09.81: Nasal congestion (21.7%) |
