# Supplementary material for: Pediatric Long COVID Subphenotypes: An EHR-based study from the RECOVER program
Source: PLOS Digit Health. 2025 Apr 10;4(4):e0000747. doi: 10.1371/journal.pdig.0000747 (PMC11984710; doi:10.1371/journal.pdig.0000747)
Supplement: S3 Table — (DOCX) [file pdig.0000747.s011.docx]

**S3 Table: Most common medications and procedures by subphenotype, cohort B**

| Subphenotype | Fatigue | Gastrointestinal symptoms | Headache | Musculoskeletal pain | Neuropsychiatric conditions | Respiratory/cardiac symptoms |
| --- | --- | --- | --- | --- | --- | --- |
| Most common procedures | 93005: Electrocardiogram, routine ECG with at least 12 leads; tracing only, w… (24.3%)  93010: Electrocardiogram, routine ECG with at least 12 leads; interpretation … (21.1%)  36415: Collection of venous blood by venipuncture (16.3%)  71046: Radiologic examination, chest; 2 views (14.7%)  93306: Echocardiography, transthoracic, real-time with image documentation (2… (14.3%) | 43239: Esophagogastroduodenoscopy, flexible, transoral; with biopsy, single o… (20.9%)  36415: Collection of venous blood by venipuncture (18.0%)  74018: Radiologic examination, abdomen; 1 view (16.1%)  76705: Ultrasound, abdominal, real time with image documentation; limited (eg… (15.1%)  76700: Ultrasound, abdominal, real time with image documentation; complete (11.4%) | 36415: Collection of venous blood by venipuncture (15.3%)  70551: Magnetic resonance (eg, proton) imaging, brain (including brain stem);… (14.7%)  97110: Therapeutic procedure, 1 or more areas, each 15 minutes; therapeutic e… (11.1%)  93005: Electrocardiogram, routine ECG with at least 12 leads; tracing only, w… (10.8%) | 97110: Therapeutic procedure, 1 or more areas, each 15 minutes; therapeutic e… (27.3%)  97161: Physical therapy evaluation: low complexity, requiring these component… (20.1%)  97112: Therapeutic procedure, 1 or more areas, each 15 minutes; neuromuscular… (12.2%) 36415: Collection of venous blood by venipuncture (10.2%) | 36415: Collection of venous blood by venipuncture (14.2%) | 71046: Radiologic examination, chest; 2 views (17.5%)  93005: Electrocardiogram, routine ECG with at least 12 leads; tracing only, w… (17.3%)  93010: Electrocardiogram, routine ECG with at least 12 leads; interpretation … (16.4%)  36415: Collection of venous blood by venipuncture (11.4%)  93306: Echocardiography, transthoracic, real-time with image documentation (2… (11.4%) |
| Most common medications | 745678: albuterol Metered Dose Inhaler (11.6%) | 373149: ondansetron Disintegrating Oral Tablet (21.4%)  876077: calcium chloride / lactate / potassium chloride / sodium chloride Inje… (20.4%)  1870358: polyethylene glycol 3350 Powder for Oral Solution (19.3%)  376327: ondansetron Injectable Solution (18.9%)  378236: omeprazole Delayed Release Oral Capsule (17.2%) | 370674: ibuprofen Oral Tablet (12.1%)  370570: amitriptyline Oral Tablet (11.8%)  373149: ondansetron Disintegrating Oral Tablet (10.7%) |  |  | 745678: albuterol Metered Dose Inhaler (16.5%)  370672: ibuprofen Oral Suspension (11.4%)  876077: calcium chloride / lactate / potassium chloride / sodium chloride Inje… (10.5%)  370509: acetaminophen Oral Suspension (10.5%) |
